# Wild Zebrafish Sentinels: Biological Monitoring of Site Differences Using Behavior and Morphology

**DOI:** 10.3390/toxics9070165

**Published:** 2021-07-12

**Authors:** Jeffrey R. Kelly, Sierra G. Shelton, Danita K. Daniel, Anuradha Bhat, Rubina Mondal, Fahren Nipple, Halima Amro, Myra E. Bower, Gabriel Isaac, Gillian McHaney, Emilia P. Martins, Delia S. Shelton

**Affiliations:** 1 Department of Psychology, University of Tennessee, Knoxville, TN 37996, USA; jkelly56@vols.utk.edu (J.R.K.); hamro@vols.utk.edu (H.A.); mbower1@vols.utk.edu (M.E.B.); gisaac@vols.utk.edu (G.I.); gmchaney@vols.utk.edu (G.M.); 2 School of Business, Stillman College, Tuscaloosa, AL 35401, USA; sierragrace.shelton@gmail.com; 3 Department of Biological Sciences, Indian Institute of Science Education and Research Kolkata, Mohanpur 741246, India; Danita.daniel@gmail.com (D.K.D.); anuradhabhat@iiserkol.ac.in (A.B.); rubinamondal55@gmail.com (R.M.); 4 Department of Chemistry and Forensic Science, Albany State University, Albany, GA 31705, USA; fnipple@umaryland.edu; 5 School of Life Sciences, Arizona State University, Tempe, AZ 85281, USA; Emilia.Martins@asu.edu; 6 Environmental and Molecular Toxicology, Sinnhuber Aquatic Research Laboratory, Oregon State University, Corvallis, OR 97333, USA

**Keywords:** environmental change, wild zebrafish, danio rerio, sentinel, behavior, morphology, India

## Abstract

Environmental change poses a devastating risk to human and environmental health. Rapid assessment of water conditions is necessary for monitoring, evaluating, and addressing this global health danger. Sentinels or biological monitors can be deployed in the field using minimal resources to detect water quality changes in real time, quickly and cheaply. Zebrafish (*Danio rerio*) are ideal sentinels for detecting environmental changes due to their biomedical tool kit, widespread geographic distribution, and well-characterized phenotypic responses to environmental disturbances. Here, we demonstrate the utility of zebrafish sentinels by characterizing phenotypic differences in wild zebrafish between two field sites in India. Site 1 was a rural environment with flowing water, low-hypoxic conditions, minimal human-made debris, and high iron and lead concentrations. Site 2 was an urban environment with still water, hypoxic conditions, plastic pollution, and high arsenic, iron, and chromium concentrations. We found that zebrafish from Site 2 were smaller, more cohesive, and less active than Site 1 fish. We also found sexually dimorphic body shapes within the Site 2, but not the Site 1, population. Advancing zebrafish sentinel research and development will enable rapid detection, evaluation, and response to emerging global health threats.

## 1. Introduction

Environmental change is rapidly accelerating, producing profound changes in natural environments [1,2,3,4,5]. Aquatic ecosystems are changing at alarming rates due to anthropogenic influences including temperature, carbonization, acidification, flow, and pollution [6,7,8,9,10,11]. These changes produce devastating effects on ecosystems [12,13,14] and human health [15,16,17]. Marginalized communities are disproportionately impacted by environmental change [18,19,20]. The severity and rapid change in aquatic ecosystems requires real-time assessments in the field using sentinels to characterize site conditions [21]. Zebrafish (*Danio rerio*) can serve as effective biological monitors for site differences because of their widespread distribution [22] and technical innovation [23].

Ecosystems are differentially impacted by environmental change due to the diversity of environmental conditions among regions [24,25,26]. Current methods for assessing environmental change include measuring water parameters, adaptation and evolution among local organisms, and species abundance across temporal scales [27,28,29,30,31]. Although these metrics provide insights into the impact of environmental change on the locale or site, they often necessitate meticulously trained personnel, expensive techniques and time-consuming laboratory procedures. Such requirements lead to exclusive technologies that are impractical for detecting rapid, emerging changes in environments, and, consequently, exacerbate environmental health disparities [32,33,34,35]. It is necessary to characterize a sentinel species that will provide powerful and quick assessments of environmental conditions that are accessible to non-specialists and individuals seeking alternative monitoring methods.

Many aspects of environmental change including temperature, oxygenation, water flow, and pollution alter morphology and behavior in multiple taxa [36,37,38,39,40,41,42,43,44,45,46]. Quantification of these morphological and behavioral endpoints in the lab and in the field can thus be utilized to expeditiously detect differences in environmental conditions across sites using a sentinel species. Analysis of organism morphology and behavior can be accomplished quickly and inexpensively by both specialists and non-specialists alike to characterize site differences in real-time. Zebrafish morphology and behavior vary across these endpoints and can be used as a proxy to rapidly detect these changes in environmental conditions [47,48,49,50,51,52]. Zebrafish are an ideal sentinel species for detecting site differences across these endpoints because they eliminate the need for cost-prohibitive laboratory technology and assays, provide a unique opportunity for use in lower socio-economic regions, and serve as alternatives for communities seeking disruptive technologies [34].

Zebrafish are powerful model organisms utilized in multiple disciplines due to their vast biomedical toolkit and well-characterised phenotypic and genotypic changes in response to environmental change [49,53,54,55,56]. The use of zebrafish as laboratory-based sentinels for toxicity has been embraced internationally through the development of the International Organization for Standardization Zebrafish Toxicity Test [57], the British standard BS/EN/ISO 7346-3-1998, the German standard DIN/EN/ISO 7346-3-1998, and the Chinese standard GB/T13267-91 [53,58]. Zebrafish are found in rivers, streams, canals, and rice paddies of India, Pakistan, Bangladesh, Nepal, Myanmar, and Bhutan [59]. Feral zebrafish distribution includes Brazil, Colombia, Malaysia, Sri Lanka, Thailand, and the United States [60,61,62,63,64,65,66]. Domestic zebrafish are found in over 100 countries in more than 3000 institutions [22], and are a popular aquarium fish. Through escape, release, and reproductive fervor, zebrafish may have an even larger distribution. Due to their wide distribution across rural and urban environments, zebrafish are present in regions with drastically different environmental conditions, and can be leveraged in these environments to characterize site differences. Environmental change disproportionately affects marginalized people, so deploying accessible biological monitors is needed [18,19,20]. The use of wild zebrafish as sentinels for environmental change will thus be a crucial step in improving the health of marginalized communities, leading to global health improvements for all.

Here, we present simple morphometric and behavioral measures that can be conducted at various field sites by both specialists and non-specialists to detect local site differences using zebrafish. All analyses can be performed rapidly in real-time using free and open-source software, allowing for expeditious assessment and minimal financial burden on investigators. Our goal is that these assays be used for community scientists living where zebrafish are found to facilitate the evaluation of environments and to guide decisions to improve health outcomes. To highlight the utility of wild zebrafish as sentinels using these proposed metrics, we characterized phenotypic differences in zebrafish sampled from two Indian populations that differed in environmental conditions. One population (Site 1) was obtained from a rural environment with flowing water with low-hypoxic conditions that had minimal human-made debris and iron and lead concentrations that exceeded acceptable limits, and the other population (Site 2) was from an urban environment with still water with hypoxic conditions, plastic pollution, and arsenic, iron, and chromium concentrations that exceeded acceptable WHO and BIS arsenic limits [27,67,68,69]. To assess phenotypic differences between these populations, we used three classes of endpoints: body shape, social behavior, and activity. We propose that these three endpoint classes monitored in wild zebrafish can be used as indicators to detect site differences to rapidly characterize the impacts of environmental change.

## 2. Materials and Methods

### 2.1. Study Location & Subjects

We conducted a field study at two field sites in India during the post-monsoon season in November 2017. Wild zebrafish were previously studied at these field sites [49,70,71,72]. The sites differed in documented pollution, human population density, oxygenation, and water flow (Table 1).

We sampled zebrafish from a rural site (Site 1), a fast-moving river in West Bengal, northeast India (a tributary of the Torsa River in Cooch Behar) with low normoxic conditions. Site 1 had arsenic concentrations below (0.01 mg/L), iron (0.3 mg/L) and lead (10 µg/L) concentrations above the WHO and BIS acceptable limits, and was situated in a location with a low human population density (~700 persons/km²) [27,73]. The other population of zebrafish was sampled at an urban site (Site 2), a canal with stagnant water in the Nadia district of West Bengal, a densely populated human settlement (~1250 persons/km²) with hypoxic conditions, and arsenic, iron, and chromium (0.05 mg/L) concentrations that exceeded the WHO and Indian Standard acceptable limits [27,52,74,75,76,77,78]. It should be noted that BIS, but not the WHO, has limits on iron in drinking water. At all sites, the fish were collected by local fishermen in the morning. The fish were photographed for morphometric analysis and placed in a holding container with local water for at least an hour prior to the commencement of preparation for behavioral trials. A total of 144 zebrafish were sampled, with 72 fish from each field site, a larger sample size than in other recent zebrafish field studies [52,71]. A random collection of the fish led to sampling 62 male fish and 9 female fish from Site 1, and 49 male fish and 23 female fish from Site 2. All fish were visually sexed prior to analysis [60]. One Site 1 fish was omitted from morphometric analysis due to a missing photograph, decreasing the total sample size for morphometric analysis to n = 143.

### 2.2. Ethics Statement

All procedures involving animals were approved by Arizona State University’s Institutional Animal Care Use Committees under protocol 17-1596R on 22 June 2017, and complied with the existing rules and guidelines outlined by the Committee for the Purpose of Control and Supervision of Experiments on Animals (CPCSEA), Government of India, the Institutional Animal Ethics Committee (IAEC), and the Indian Institute of Science Education and Research (IISER) Kolkata.

### 2.3. Data Collection & Analysis

Following a post-capture acclimation period of at least 1 h, we divided zebrafish into equal groups of 6 fish, creating 12 groups per field site. Before behavioral testing, we photographed all fish from above. All photos were taken at the same height and with a scale within the frame to allow for morphological analysis. We then sequentially placed zebrafish into a white plastic tank (dimensions 30.8 cm × 34 cm) filled to a height of 5 cm with habitat water, where they acclimated for an additional 5 min before behavioral assessments, giving a total acclimation time of 65 min. The tanks were recorded from above using a GoPro Hero 5 Session camera. The acclimation period appeared sufficient as the fish did not freeze and displayed exploratory behavior consistent with an absence of stress responses [80,81].

We extracted behavioral data from video recordings using ToxTrac video tracking software in a 3 min sample period [82,83]. We identified fish within the tank by adjusting contrast and size thresholds for detection. A minimum threshold tracking accuracy of 80% was required to consider video tracking reliable. This threshold was determined based on the minimum Kappa statistic of inter-rater reliability for data to be deemed reliable [84]. We analyzed all data using RStudio [85,86].

We categorized video data as either Social measures or Activity measures. Social measures analyzed were inter-individual distance (average distance from each individual to all other group members), time in proximity (number of seconds spent within two body-lengths of a group member), and nearest-neighbour distance (average distance to the closest group member). As the threshold for size detection for video tracking was most reliable between 400–500 pixels for both groups, we standardized the distance used for the two-body-length metric as 1000 pixels. Pixel data were converted to millimeters for statistical analysis. Activity measures analyzed were distance traveled (total number of mm traversed), swim speed (the number of mm traversed divided by the sample duration), tank exploration (mm² of the tank visited), and number of seconds in motion. We collected data for each individual and collapsed data into tank means for analysis between levels of Population to account for the independence of observations, leaving a total of 12 observations per field site. As residuals were normally distributed we analyzed behavioral data with Welch’s two-sample *t*-tests between populations.

**Figure 1:**
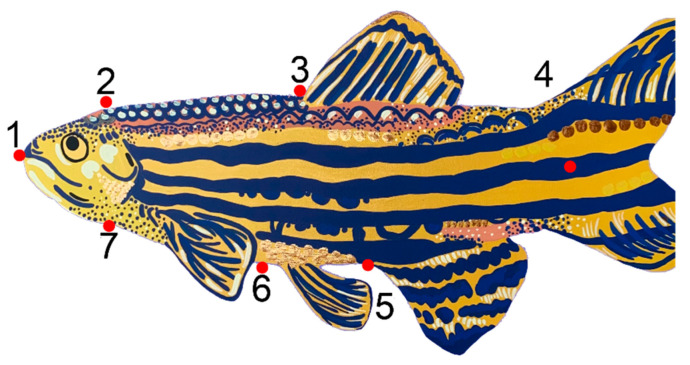
A total of seven digitized landmarks were placed on distinct points of zebrafish for use in the morphometric analysis. Landmarks denote: (1) mouth, (2) posterior dorsal skull, (3) anterior dorsal fin, (4) caudal fin, (5) anterior anal fin, (6) ventral surface, and (7) ventral anterior skull. Art created by Gillian McHaney.

We collected morphometric and geomorphometric data using the TPS series software [87]. We placed seven landmarks on each photograph of subjects to capture the shape of each individual using tpsUtil [88] and tpsDIG [89] (Figure 1). We based our selection of landmarks on previous studies that characterized zebrafish body length, depth, and shape in the field and in toxicology studies [52,71,90,91]. We reduced the number of landmarks to make the endpoint amenable to high-throughput assessments. We computed partial warps and consensus shapes for all individuals using the seven digitized landmarks with tpsRelw [87,92]. We analyzed the effects of independent variables on fish shape using a multivariate analysis of variance (MANOVA). These shape variances were explored by comparing the relative size ratios between these regions across levels of Population and Sex with a two-way analysis of variance (ANOVA). As residuals did not meet the assumption of normality, total posterior, and anterior body region size was contrasted across all classes of individuals using Wilcoxon Signed Rank paired *t*-tests. Finally, we calculated standard length as the distance between landmarks 1 and 4 (length from the anterior to posterior points of the body) and analyzed length data with a Kruskal Wallis test. All morphometric analyses were conducted using the geomorph [93] and lme4 [94] packages.

## 3. Results

### 3.1. Fish Body Shape Is Distinct between Sites

**Figure 2:**
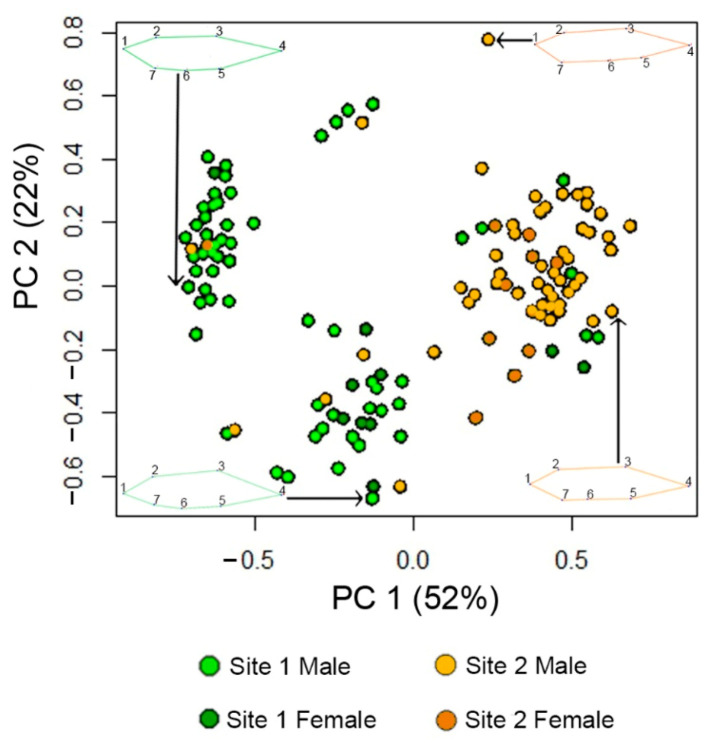
Procrustes PCA plot of variance in partial warps between levels of Population and Sex. The first two Principal Components account for 74% of the variance in consensus shape between populations. The landmarks of specimens at the negative and positive ends of the first and second axes are shown not scaled to size. Site 1 fish are labeled in green, and Site 2 fish are labeled in orange. Lighter shades denote male fish and darker shades denote female fish.

We found distinct fish shape differences between the two populations. We analyzed partial warps of fish using PCA and found that the first two axes explained 74.69% of the total variance in body shape (Figure 2). Morphometric differences between Population and Sex levels were plotted using composite plots of consensus shape (Figure 2). We then compared these morphometric differences across levels of Population and Sex using MANOVA to find statistical differences in consensus shape. We found a significant main effect of Population on consensus shape (*F*(1140) = 3.44, *p* = 0.001), as well as a non-significant trend for an interaction between Population and Sex (*F*(1140) = 1.137, *p* = 0.07). Consensus shape differed between sexes, but did not reach statistical significance, *F*(1140) = 0.872, *p* = 0.19. To characterize these differences in morphology between populations, we then analyzed body size ratio and standard length.

**Figure 3:**
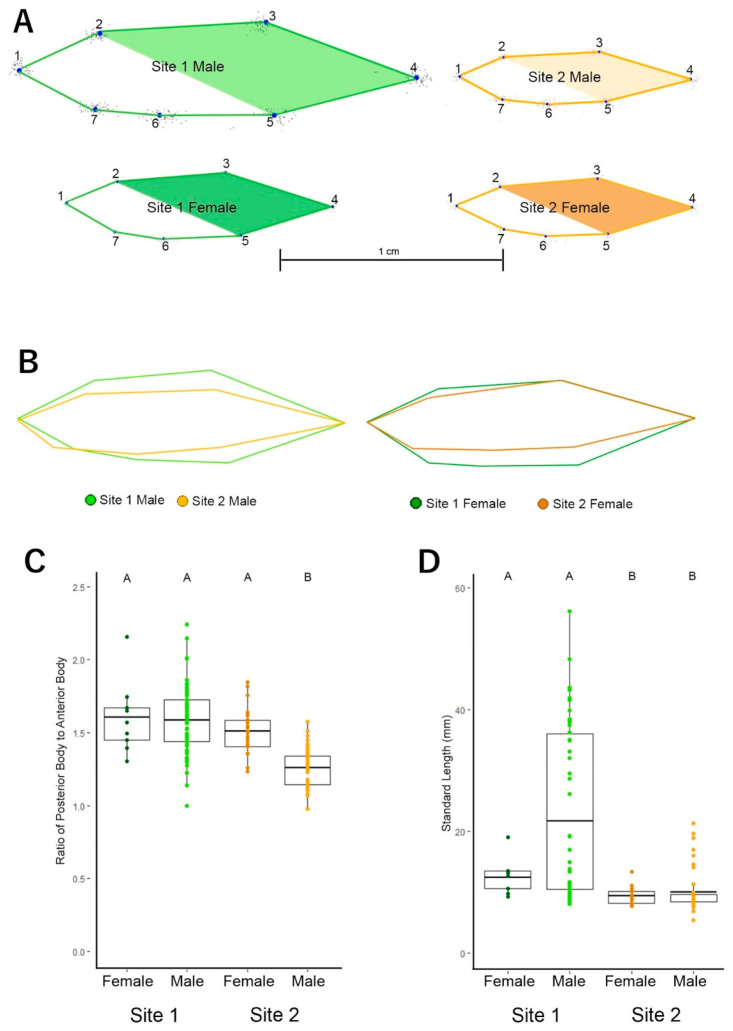
Consensus shape differed significantly across Population, but not Sex. The greatest variance in shape between populations was among landmarks located on the posterior dorsal skull (LM 2), anterior dorsal fin (LM 3), caudal fin (LM 4), and anterior anal fin (LM 5). (**A**) Size differences in fish between levels of Population and Sex. Shaded regions denote the posterior body region identified by PCA as contributing to shaping variance, while the white region denotes the anterior body region not identified by PCA. (**B**) Overlaid shapes of the most morphometrically-extreme fish across levels of Population and Sex. (**C**) Fish from Site 1 and Site 2 female fish had a larger posterior region of their body relative to the anterior region compared to Site 2 male fish. Individual samples are overlaid onto boxplots. Data analyzed with a two-way ANOVA. (**D**) Fish from Site 1 had a greater standard length than fish from Site 2. Statistical analysis showed that Site 2 fish, not Site 1 fish, were sexually dimorphic. Individual samples are overlaid onto boxplots. Data analyzed with a Kruskal-Wallis test. Boxplots denote means and interquartile range. Error bars indicate the range of scores. Letter markings donate significant (different letters) and non-significant (same letters) differences (*p* < 0.05).

We compared the relative area of the posterior body region (LM 2, 3, 4, 5) to the anterior body region (LM 1, 5, 6, 7) (Figure 3). The posterior region of all fish was significantly larger than their anterior regions (Site 1 Female Anterior M = 97.3 mm² ± 14.31, Posterior M = 153.4 mm² ± 22.86 SEM; Site 1 Male Anterior M = 429.7 mm² ± 59.69 SEM, Posterior M = 676.9 mm² ± 96.83 SEM; Site 2 Female Anterior M = 55.2 mm² ± 3.14 SEM, Posterior M= 83.5 mm² ± 5.45 SEM; Site 2 Male Anterior M = 77.1 mm² ± 8.176 SEM, Posterior M = 96.7 mm² ± 10.62 SEM; all *p* < 0.0001). Further analysis showed that the ratio between posterior and anterior body regions of Site 2 male fish (M = 1.3, SEM = 0.02) was 22% smaller than Site 1 female fish (M = 1.6, SEM = 0.08), 21% smaller than Site 1 male fish (M = 1.6, SEM = 0.03), and 17% smaller than Site 2 female fish (M = 1.5, SEM = 0.03) (Figure 3). This difference led to a significant two-way interaction between levels of Population and Sex on body size ratio, *F*(1, 140) = 7.79, *p* = 0.01. When analyzed with Tukey’s post-hoc test, we found that all fish had relatively larger posterior regions than Site 2 male fish (Site 1 Female vs. Site 2 Male *p* < 0.0001, Site 1 Male vs. Site 2 Male *p* < 0.0001, Site 2 Female vs. Site 2 Male *p* < 0.0001). This shows that Site 1 fish and Site 2 female fish have a significantly more torpedo shape with elongated posterior ends than male fish from Site 2 [95].

Male fish from Site 1 were the longest fish (M = 2.2 cm, SEM = 0.18), measuring more than twice as long as both male (M = 1.0 cm, SEM = 0.05) and female (M = 0.9 cm, SEM = 0.03) fish from Site 2, and nearly twice as long as female fish from Site 1 (M = 1.3 cm, SEM = 0.10). These differences in standard length led to a significant main effect of Population on standard length, *H*(78) = 101.15, *p* = 0.040 (Figure 3).

### 3.2. Site 2 Fish Are More Cohesive Than Site 1 Fish

**Figure 4:**
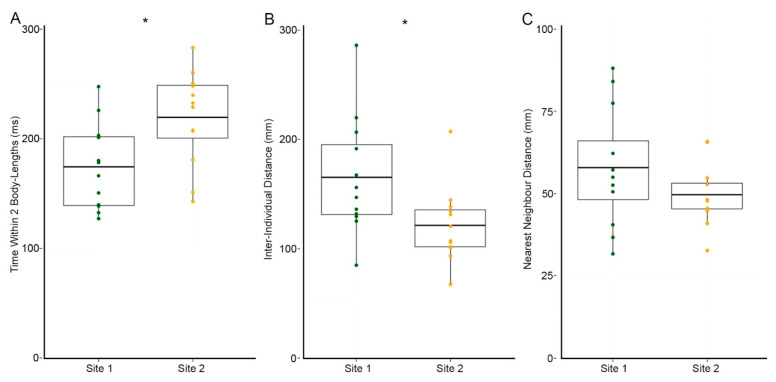
We found that fish from Site 2 were more cohesive than fish from Site 1 across three social metrics. (**A**) Fish from Site 2 spent a greater amount of time within two body-lengths of conspecifics than fish from Site 1. (**B**) Fish from Site 2 maintained closer inter-individual distances between group members, while fish from Site 1 had greater spacing between individuals. (**C**) There were no significant differences in distances between individual fish and their nearest neighbors between populations. Individual samples are overlaid onto boxplots. Boxplots denote means and interquartile range. Error bars indicate the range of scores. * *p* < 0.05. All data analyzed with independent samples *t*-tests.

Overall, we found that groups of fish from Site 2 were more cohesive than groups of fish from Site 1. Fish from Site 2 spent approximately 20% more time within 2 body-lengths of tankmates than fish from Site 1. This difference between sites led to a significant difference in time within two body-lengths, *t*(22) = −2.69, *p* = 0.014 (Site 2 M = 219.4 ms ± 12.45 SEM, Site 1 M = 174.2 ms ± 11.26 SEM) (Figure 4). Fish from Site 2 also maintained approximately one-quarter shorter inter-individual distances than fish from Site 1, *t*(22) = 2.384, *p* = 0.028 (Site 2 M= 121.1 mm ± 10.09 SEM, Site 1 M = 165.1 mm ± 15.49 SEM) (Figure 4). We found a similar pattern of increased cohesion in Site 2 using the Nearest Neighbor Distance metric, though this finding did not reach statistical significance, *t*(22) = 1.42, *p* = 0.175 (Site 1 M = 165.1 mm ± 15.49 SEM, Site 2 M = 121.1 mm ± 10.09 SEM) (Figure 4).

### 3.3. Site 1 Fish Are More Active Than Site 2 Fish

**Figure 5:**
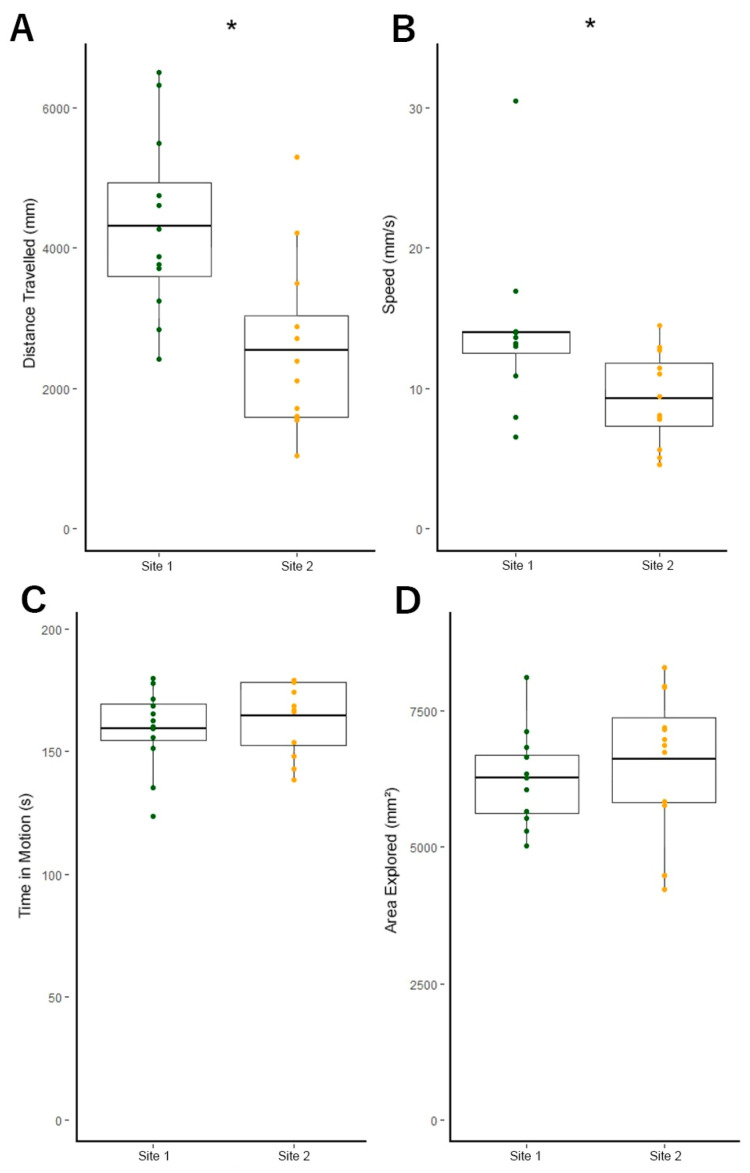
Site 1 fish swam faster and further, but fish from both populations maintained similar time in motion and exploration. (**A**) Fish from Site 1 swam significantly greater distances than fish from Site 2. (**B**) Fish from Site 1 exhibited significantly greater average swimming speeds than Site 2. (**C**) Fish from each population spent a similar amount of time in motion throughout the sample period. (**D**) Fish from each population explored a similar number of areas within the testing arena and thus did not differ significantly in exploratory behavior. Individual samples are overlaid onto boxplots. Boxplots denote means and interquartile range. Error bars indicate the range of scores. * *p* < 0.05. All data analyzed with independent samples *t*-tests.

We also found that fish from Site 1 were more active than fish from Site 2. Fish from Site 2 swam at two-thirds the speed as fish from Site 1, *t*(22) = 2.42, *p* = 0.027 (Site 1 M = 13.9 mm/s ± 1.71 SEM, Site 2 M = 9.3 mm/s ± 0.95 SEM) (Figure 5). Similarly, fish from Site 1 swam nearly twice as far on average than fish from Site 2, *t*(22) = 3.40, *p* = 0.003 (Site 1 M = 4316.9 mm ± 372.09 SEM, Site 2 M = 2547.1 mm ± 363.56 SEM) (Figure 5). We found that fish from both populations spent a similar amount of time in motion (*t*(1,22) = −0.82, *p* = 0.423, Site 1 M = 159.3 s ± 4.74 SEM, Site 2 M = 164.5 s ± 4.29 SEM) (Figure 5), with no observed instances of any fish freezing in the tanks. Exploration was not affected by swimming speed or distance traveled, as both populations explored the majority of the testing arena and did not differ significantly in the area of the tank explored (*t*(22) = −0.79, *p* = 0.44, Site 2 = 69.7% explored ± 3.96 SEM, Site 1 = 65.9% explored ± 2.59 SEM) (Figure 5).

As there were disproportionately more male than female fish sampled, we investigated whether behavior across these levels differed between single-sex and mixed-sex groups. We found that fish in mixed-sex groups from both populations swam nearly half the distance as single-sex groups (Mixed-Sex M = 2648.0 mm ± 278.22 SEM, Single-Sex M = 4738.7 mm ± 439.51 SEM, *F*(1,20) = 7.06, *p* = 0.02). Mixed- and single-sex groups did not significantly differ across other metrics. However, the Site 2 sample had 11 mixed-sex tanks and 1 single-sex tank while the Site 1 sample had 4 mixed-sex tanks and 8 single-sex tanks. Thus, the difference in distance travelled may reflect population, but not group composition-dependent activity.

## 4. Discussion

We propose three endpoints that can be used in wild zebrafish to detect site differences. Morphometric, Social, and Activity endpoints aid in characterizing phenotypic differences between wild populations of zebrafish from sites with differing environmental conditions. Zebrafish showed between-population and sex differences in size, with longer Site 1 fish than Site 2 fish (Figure 3). We found sexual dimorphism for body size ratio in Site 2; Site 2 male fish had more similar posterior and anterior body regions, leading to a smaller body size ratio, whereas Site 2 female fish had larger anterior regions and smaller posterior regions resulting in a larger body size ratio. The Site 1 fish had posterior body regions that dwarfed the anterior body regions (Figure 3). There was a visible sexual dimorphism for length within Site 1; however, this did not reach statistical significance, likely because of the low number of female fish and high male size variability (Figure 3). We found that fish from Site 2 were more cohesive than Site 1 fish, spending more time within two body-lengths and maintaining closer inter-individual distances between group members. Fish from Site 1 were more active, swimming faster and further distances than Site 2, more polluted fish. Our results indicate that these measurements can be used to identify site differences. Given that there is a relationship between reported site-specific environmental conditions and wild zebrafish morphology and behavior, an intensive research program is needed to identify the causal relationship between the phenotypic differences and pollutants, and other abiotic and biotic factors.

Phenotypic variation across these endpoints in zebrafish has been shown in laboratory settings previously. Zebrafish exposed to plastic pollution [96,97], arsenic [98], lead [99], antibiotics [100], polycyclic aromatic hydrocarbons [101], organic pollutants [102], elevated temperatures [103], or emerging pollutants [104] may exhibit altered body mass, size, and swimming behavior. Similarly, hypoxia [52,105] and water flow [49,52,70,71] are associated with changes in zebrafish morphological and behavioural phenotypes. Other studies documented larger fish with deeper bodies and caudal peduncles in flowing water, and relatively dwarfed regions in fish from stagnant water, similar to the present study (Figure 2 and Figure 3); sites sampled in the previous studies overlapped with the present study [52,71]. Hypoxic conditions [106], sex-specific predation pressure [107], or endocrine disruptors [108] and other pollutants [109] might have also led to the male-biased sex ratio in these populations. Our findings show these measurements can be used in the field to assess population differences. The observed phenotypic variation across a range of environmental factors demonstrates the utility of zebrafish biological monitors. The zebrafish’s broad rangeland coupled with its significance in biomedical research leverages its candidacy as a potential flagship umbrella species, or through their socio-ecological capital and conservation, zebrafish can extend protection to others [110,111]. To effectively develop zebrafish as sentinels there is a need for tight integration of lab and field studies.

Using wild zebrafish as sentinels for site differences provides unique benefits, including rapid assessment, interrogation of diverse, novel chemical profiles, and a cost-effective approach. Laboratory analysis of environmental conditions can take extensive periods to complete, and is frequently limited to assessing a small number of factors which decreases external validity [112,113,114]. We suggest zebrafish as sentinels that can harmonize sampling and analysis of environmental conditions. Zebrafish’s small size, adaptability, and presence near the surface in clear water permits the positioning of accessible, inconspicuous monitoring stations in areas of interest, such as near residential areas or regions with factory or agricultural activity [53,70]. Zebrafish’s biomedical toolkit, rapid generation time, well-characterized behavioral repertoire, evolutionarily conserved traits and genetic homology across vertebrates permit advances in mechanistic, life span, and predictive toxicology and conservation [115,116,117,118,119]. Implementing zebrafish as biological monitors increases the potential to rapidly detect, evaluate, and respond to environmental change, thus facilitating preservation, interventions, bioremediation, and improvement of health outcomes.

The next steps required for implementing zebrafish sentinels are comprehensive and predictive characterization of phenotypic variation in response to different classes and combinations of biotic and abiotic factors. This could be accomplished by analyzing zebrafish phenotypes at multiple field sites, supplemented with identifying and quantifying factors associated with environmental change tested in a laboratory, and predictive modeling [120]. Due to the increased complexity of field versus laboratory environments, it is imperative to validate that observed phenotypic variation results from hypothesized sources of variation rather than external variables such as water flow, predation, environmental complexity and stability, genetics, and social context [49,52,70,71,72,121,122]. Optimization and implementation of zebrafish biological monitors in the field could rely on partially-submerged slotted tanks or cages with passages for water flow but not fish and with grated lids to prevent predation. Using laboratory strains of zebrafish and common garden or transplant designs will increase monitoring sensitivity by minimizing potential influences of developmental or evolutionary adaptation to water conditions [123,124,125]. Studies should house fish in same-sex groups, or utilize sterile or triploid fish to prevent disruption of natural ecosystems through the breeding of domestic and wild fish [126,127]. Genetic or physiological identification techniques such as fluorescent tagging can also ensure monitoring fidelity and experimental integrity. Many of these technologies exist in disparate fields and have the potential to be realized through Collaborative, Entrepreneurial, Translational, and Innovative (CETI) research and development.

### Additional Thoughts on Using Wild Zebrafish as Sentinels

Just, Equitable, Diverse, and Inclusive (J.E.D.I.) research practices are required to effectively implement biological sentinels [128,129,130,131]. Just sentinel programs require developers to center environmental justice and racism at the forefront by recognizing environmental change risks are unequally distributed by race and class [32,33,132]. Monitoring systems and procedures should be accessible and Equitable, so that members of local communities with varying financial, natural, produced, and human capital can obtain sampling systems to quickly and actively monitor their environments [133]. For example, people and communities whose capital is devoid of zebrafish (i.e., zebrafish are absent), knowledge of fish, or physical environments conducive to using the monitoring device (e.g., concrete city landscapes), equitable technologies, training, and surrogate species should be implemented. Diverse endpoints, at multiple timepoints in several populations in the lab and field, can aid detection, assessment, and causal inference, thereby providing a more holistic view of environmental change. Simplifying assessments will permit endpoints that rely on single axes that accurately distinguish male vs. female [134], and exposed vs. unexposed [90] zebrafish at different developmental exposure periods [135], thereby dramatically enhancing screening throughput. Using a breadth of biomarkers at multiple time points is essential for detecting adverse effects because some deleterious perturbations are revealed at some endpoint, but not others [136]. Inclusive sentinel programs involve local people and communities as contributors and decision-makers in developing the biological monitors. Including users as decision makers and developing technology that addresses their pains is common in startups prioritizing customers [137].

Startups are engines of ingenuity that can help introduce zebrafish sentinel devices into the market as commercial tools [138]. Entrepreneurial thinking may accelerate zebrafish sentinel monitors into disruptive technologies, or technologies that are cheaper, simpler, and more convenient than the dominant technology, thereby leading to an upheaval in the existing market structure [34]. Prioritizing sentinels to help people combat environmental racism may provide a competitive advantage, because it taps a market that has cost the USD 16 trillion in revenue over the last 20 years [139]. Evaluating the implementation of zebrafish sentinels and competing technologies is needed to determine their impact and ability to enhance human and environmental health. Some dimensions of evaluation may include changes in health disparities, environmental engagement, stewardship and community resilience [140,141]. Zebrafish sentinels can be technological innovations that may lead to socio-economic-environmental-political changes in society.

## 5. Conclusions

Environmental change poses a considerable threat to natural environments and global health [1,2,3,4,5]. Using wild zebrafish as sentinels is an efficient and economical approach to monitoring, assessing and addressing global health risks. Developing zebrafish as sentinels in the field fills the need for rapid water quality analysis not addressed by laboratory-based methods, and presents a unique opportunity for implementation in underserved regions and communities looking for alternative monitoring systems. Utilizing social, activity, and morphometric endpoints in zebrafish highlighted here can thus provide a crucial step in assessing site differences. Coupling JEDI biological monitoring practices with CETI research and development will lead to technology that transforms environmental monitoring and improves health outcomes worldwide.

## Figures and Tables

**Table 1:** Description of conditions at both field sites.

Site	Site 1	Site 2
**District**	Cooch Behar	Nadia
**Pollutants**	As <0.01 mg/L [27] Iron (2.3–6.5 mg/L), Lead (0–16.6 μg/L) [27], minimal human-made debris	As, 0.1 mg/L–0.5mg/L [67,69], Iron (0–9.1 mg/L), Chromium (0–147.8 μg/L) [27], abundant human-made debris: plastic bottles, styrofoam plates, bags, shoes [52]
**Water use**	People were observed bathing, washing clothes and irrigating crops with water from the field site	People tried to minimize contact with the water. When contact was made with water people described burning and itching sensations.
**Land use**	Homes, rice cultivation, sand mining, livestock grazing, unpaved roads	Homes, buildings, shops, paved roads
**Water Flow**	Flowing [71]	Stagnant [71]
**pH**	7.4 [71]	6.9–7.8 [70,71]
**Total Dissolved Solids (ppt)**	0.2 [71]	0.3 [70,71]
**Dissolved Oxygen (ppm)**	3.2–5.4 [79]	1.5 [52]
**Nitrate (ppm)**	0.0 [70]	0.0 [70]
**Nitrite (ppm)**	0.5 [70]	0.0 [70]
**Temperature (°C)**	19–25.6 [71]	21.4–25.6 [70,71]
**Elevation (m)**	12.2 [71]	41.4 [70,71]
**Substrate**	Mud [71]	Silt [71]
**Predation Pressure**	less [71]	greater [71]
**Population Density**	~700 persons/km² [52]	~1250 persons/km² [52]

## Data Availability

Data from this publication have been archived in the Mendeley Data Repository (https://data.mendeley.com/datasets/7gbxfg7jy5/2, accessed on 9 March 2021).
